# Simulation studies of social systems: telling the story based on provenance patterns

**DOI:** 10.1098/rsos.240258

**Published:** 2024-08-07

**Authors:** Pia Wilsdorf, Oliver Reinhardt, Toby Prike, Martin Hinsch, Jakub Bijak, Adelinde M. Uhrmacher

**Affiliations:** ^1^ Institute for Visual and Analytic Computing, University of Rostock, Rostock, Germany; ^2^ School of Psychological Science, The University of Western Australia, Perth, Australia; ^3^ MRC/CSO Social and Public Health Sciences Unit, University of Glasgow, Glasgow, UK; ^4^ Department of Social Statistics and Demography, University of Southampton, Southampton, UK

**Keywords:** computational modelling, data, provenance model, social simulation

## Abstract

Social simulation studies are complex. They typically combine various data sources and hypotheses about the system’s mechanisms that are integrated by intertwined processes of model building, simulation experiment execution and analysis. Various documentation approaches exist to increase the transparency and traceability of complex social simulation studies. Provenance standards enable the formalization of information on sources and activities, which contribute to the generation of an entity, in a queryable and computationally accessible manner. Provenance patterns can be defined as constraints on the relationships between specific types of activities and entities of a simulation study. In this paper, we refine the provenance pattern-based approach to address specific challenges of social agent-based simulation studies. Specifically, we focus on the activities and entities involved in collecting and analysing primary data about human decisions, and the collection and quality assessment of secondary data. We illustrate the potential of this approach by applying it to central activities and results of an agent-based simulation project and by presenting its implementation in a web-based tool.

## 1. Introduction

**Figure 1.**
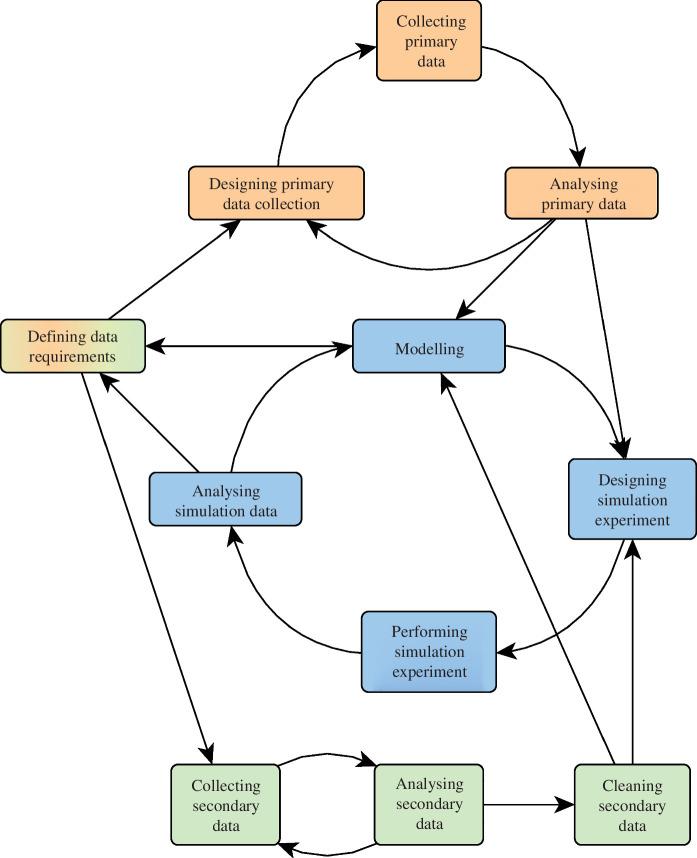
Central activities of the modelling and simulation lifecycle (blue)—including the procurement of primary data (orange) and secondary data (green).

Reproducible, traceable and understandable simulation studies require thorough documentation of activities, sources and products involved in this process [1,2]. Simulation studies involve complex modelling and analytical processes, in which activities such as model building and refinement, conducting simulation experiments (SEs), data processing and interpretation are closely intertwined (figure 1). These studies often span several years. Their documentation, therefore, requires significant effort and several reporting guidelines have been developed [1,3]. However, these are neither designed nor suited for documenting entire simulation studies, including their SEs and data collection. Furthermore, they lack a visual format suitable for inclusion in the ‘Methods’ section of a paper to illustrate the research process.

Generally speaking, all efforts in documenting simulation studies are—at least implicitly—concerned with provenance, that is providing ’information about entities, activities and people involved in producing a piece of data or thing’ [4]. The benefit of adopting a standard for provenance, such as W3C PROV (hereafter referred to as PROV) [4], is that the various sources, activities and products of a simulation study are put into well-defined relationship(s) with each other. PROV provides a historical and causal delineation of what contributed to a simulation model (SM) and how it did so in a simple and formal manner [5]. It therefore can be a formal complement to existing documentation guidelines and standards. Existing guidelines and standards mainly consist of extensive verbal descriptions and typically concentrate on specific aspects of a simulation study, such as a particular agent-based SM [6]. By contrast, PROV is capable of addressing entire simulation studies. PROV’s graph structure can be mapped into a graph database which allows—in addition to storing the information—filtering and querying the stored information on demand [7]. Its graph-based visualization (e.g. in a web-based tool) makes it possible to easily access and assess dependency structures within and across simulation studies [8]. Provenance standards have already been applied to cell biological simulation studies [8,9] and to documenting a migration model in demography [10].

In demography, as is common for social science disciplines, the need to combine various hypotheses about the mechanisms to be explored and various data sources adds to the complexity of simulation studies [11]. Thus, there is also greater effort required for their thorough and systematic documentation. The situation becomes even more complicated whenever primary data (PD) about human behaviour are collected, such as through interviews or psychological experiments conducted as part of a broader agent-based simulation study, or once evaluation schemes are used to account for uncertainty in secondary data. This is especially relevant in the context of the paradigmatic shift in demography towards more micro- and multi-level studies [12] and the recognised need for greater use of SMs to enhance the theoretical base of the discipline [13]. More broadly, this is also in line with the general developments in social simulation, which explicitly recognizes issues such as data quality and the necessity of collecting bespoke PD for simulations [14]. So far, the diversity of sources, products and processes has hampered the systematic and accessible documentation of entire demographic simulation studies.

In this paper, we present an approach based on provenance patterns specified in the PROV standard, to systematically and accessibly document entire social simulation studies, which include extensive data collection, evaluation, analysis and adaptation. In Wilsdorf *et al.* [15], provenance patterns have been identified for the documentation of and reasoning about central activities of simulation studies, such as model building and refinement, or conducting SEs for verification, calibration and validation. In the current paper, these patterns are extended to capture data evaluation schemes used to assess the quality and uncertainty of secondary data sources, and to the collection of PD, such as psychological experiments or interviews conducted, to support the agent-based modelling of human decision processes. These patterns also take reporting guidelines in the respective areas into account. To do so, we use and adapt a previously developed web-based tool for storing and retrieval of provenance information based on these patterns.

We demonstrate our approach by applying it to the development of an agent-based SM and complementary framework for assessing existing secondary data and their quality [16], as well as the acquisition of PD by conducting psychological experiments and ethnographic interviews.

The contributions of this study are the following:

- to identify key activities and entities for documenting data acquisition, quality assessment and PD collection (here, psychological experiments), and to encode them as patterns in a provenance standard;
- to integrate this meta-information with previously identified patterns for conducting simulation studies;
- to apply the patterns to the activities and results achieved within a major research project on migration to provide comprehensive documentation of the research done in this project; and
- to implement the existing and newly developed patterns for data procurement in an openly published, web-based provenance tool.

With our provenance pattern-based approach, we structure the knowledge about model building, analysis and the acquisition and use of PD and secondary data, as well as the methods of analysis. Thus, documentations based on provenance graphs can now formally represent the entire story of a simulation study (in this case, of social systems). This is an important step forward since most insights come from the relationships between artefacts, particularly the relationships with data, but also theories or specific hypotheses about the mechanisms embodied in the existing knowledge (literature). Modellers can benefit from the proposed approach by easily detecting gaps or inconsistencies in a simulation study thanks to the queryable nature of provenance graphs. The provenance information is also essential for many aspects of good scientific practice, including replicating the results, reusing SMs or interpreting the simulation results correctly in the light of available evidence. In addition, we expect the approach to be instrumental in assessing the robustness, reliability and relevance of the data collected, as well as how the insights gained from the data and the uncertainty or errors of the data collection procedure (CP) may propagate to other artefacts and processes. This can, in turn, illuminate the data and knowledge gaps, and help direct further scientific enquiry. There are known widespread issues with robustness and reliability of scientific research, such as the replication crises across psychology and other fields [17–19]. The provenance approach we propose would enable authors, reviewers and other scientists, especially coming from a diverse range of scientific disciplines, to identify scientific issues and the downstream consequences of these scientific issues (e.g. SMs that might be affected by them) more easily.

## 2. Concept

### 2.1. Provenance models and provenance patterns

**Figure 2.**
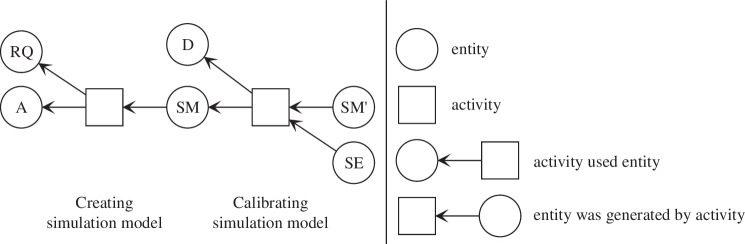
An example of a provenance graph as defined by PROV. The graph shows two typical activities in a simulation study. First, an SM is created based on a research question (RQ) and an assumption (A) about the modelled system. This produces an SM. In the next activity, the model is then calibrated against some data (D), producing a calibrated model (SM') and a specification of the performed calibration experiment (SE).

The provenance of a SM documents the process of creating the model, including: what questions it was designed to answer, on which underlying theory and data it is based, how it was constructed and how it was experimented with? This back-story of a model is crucial for interpreting and reusing a model, as well as for assessing the quality of the model and the results it generated. PROV provides a formal and standardized way to represent provenance information, i.e., ‘information about entities, activities and people involved in producing a piece of data or thing’ [4]. Following the PROV standard, provenance information can be represented as a directed acyclic graph with two types of nodes: entities and activities. These can be visually represented by ovals and rectangles, respectively. Directed edges between entities and activities relate the two (see figure 2), specifying which entities were either generated by or used by which activities. Note that in the visualizations, the edges (arrows) point back in time towards the origin of an entity or activity. Provenance graphs therefore are clearly distinguishable from flow charts, which indicate the direction of information flow.

**Figure 3.**
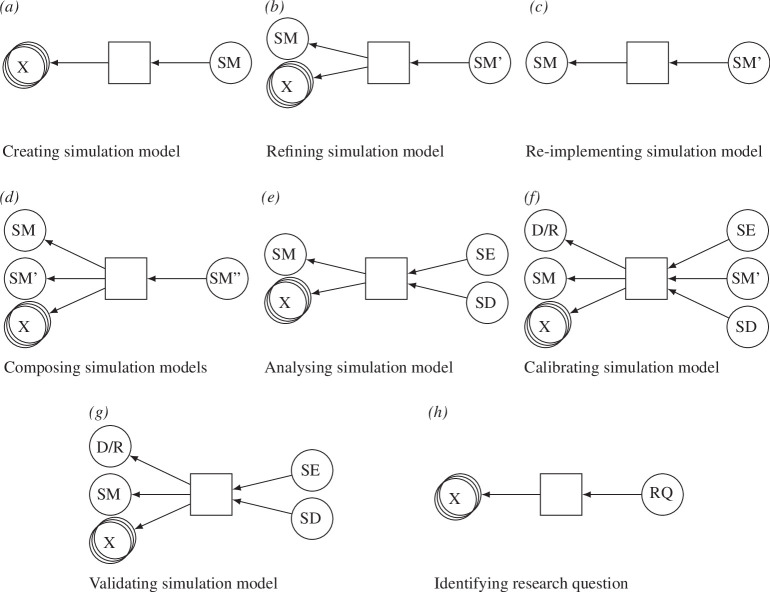
Provenance patterns for model development and model analysis activities ((*a–g*) from Wilsdorf *et al*. [15], (*h*) as introduced in this paper). The entity types involved are: SM, simulation model; SE, simulation experiment; SD, simulation data; D, data; R, requirement; RQ, research question; X, wildcard (here entities of arbitrary type can be added). SM' and SM'' denote separate entities of the type SM. There are two types of relationships: activity used entity, and entity was generated by activity.

Applying PROV requires specializing the PROV data model by specifying types of entities and activities, and possible relationships between them. For the development and analysis of SMs, the central part of a simulation study (see figure 1), important entities and activities have been identified in the literature [5,8]. Entities include SMs, experiments or research questions (RQs), whereas activities involve model building, calibration, validation and analysis. Building on this, Wilsdorf *et al*. [15] identified *provenance patterns* for model building and SEs arising in a simulation study: certain activities within a simulation study will always use and produce certain types of entities. These patterns present the fundamental constructors of a provenance graph, and also specific types of relationships to be queried. A pattern consists of an activity at its centre, and the types of entities that are used and produced by this activity. For example, figure 2 shows a provenance graph that was created by chaining two provenance patterns. The first pattern, *creating SM*, will always produce a model. The second pattern, *calibrating SM* will always use a model and a calibration target, and will always produce a new calibrated model and a specification of the calibration experiment. These and further patterns will be presented in the following (figure 3).

In a provenance graph, we can annotate entities with meta-information that contains the entities’ documentation. We recommend this meta-information follows established reporting guidelines for these types of entities or refers to a document following such guidelines, such as to an overview, design concepts and details (ODD) document for an agent-based model [20]. Additionally, the meta-information should include references to all relevant artefacts, such as the implementation of the model or the dataset for a data entity.

However, the modelling and model analysis itself, although central, is only one part of a social simulation study, which also needs to grapple with the agency of the objects under scientific investigation (human beings) and the resulting high levels of uncertainty of the related social processes. In demography, migration is the one component of demographic change which—unlike fertility or mortality—does not have explicit biological underpinnings, and is thus much more challenging to analyse owing to the high levels of agency of the various actors, and the highly complex underlying factors and drivers [21].

Another important ingredient is the data that grounds the model in reality—and the process of their collection. We distinguish PD and secondary data collection as follows:

- PD were collected specifically by the conductors of the simulation study for the simulation study itself. This collection may take the form of surveys, interviews, psychological experiments, etc; and
- secondary data were collected for another purpose, typically by someone else. Hence, its suitability must be assessed, and the data may need to be cleaned to account for various sources of uncertainty and biases.

In this work, we extend the method of provenance patterns to consider the data-related processes. In the following, we summarize the entities and activities and the arising patterns in modelling and model analysis, as defined in Wilsdorf *et al.* [15], and introduce an additional pattern for identifying RQs. Building on that, we then extend the scope of the approach by identifying entities, activities and patterns for PD and secondary data collection. Thereafter, we discuss the actors involved in conducting a social simulation study as a crucial part of provenance.

### 2.2. Modelling and model analysis

Wilsdorf *et al*. [15] mapped the considerations of existing reporting guidelines (e.g. [2,6]) into the provenance standard PROV, by identifying the central activities of a simulation study. They distinguish the following entities in the modelling and analysis part of the study; RQ, SM, SE specification, simulation data (SD), requirement (R), assumption (A) and other (O). They also make use of an entity type data (D), as a general placeholder for any kind of data, including SD produced in an SE. In our extension of the provenance patterns, data may also capture PD and secondary data (which we otherwise distinguish owing to different documentation requirements). We also extend the usage of RQs as they may provide the motivation for primary and secondary data collection and thus appear in other parts of the simulation study.

Figure 3 shows the patterns graphically. Patterns in figure 3*a-d* describe activities in the modelling process. When a new SM is created from scratch, the pattern creating SM (figure 3*a*) applies. That activity uses various inputs, e.g. an RQ, assumptions, theories or data, represented by the wildcard (X) in the pattern, and produces an SM. When an existing model is refined, the pattern refining SM (figure 3*b*) applies instead, which has an additional input in the form of the existing SM. For example, the model from the case study was refined when new data from psychological experiments became available and could be used to improve the decision-making mechanisms. To denote different versions of an artefact, the prime symbol is used, see M’ and M’ in figure 3*b,c,d,f*. The pattern re-implementing SM (figure 3*c*) refers to an activity, where an SM is re-implemented in another language or tool, without refining or extending it. This pattern may be used when cross-checking two models (e.g. [22]). Finally, the pattern composing SMs (figure 3*d*) describes the composition of two SMs.

The patterns in figure 3*e-g* describe activities during the analysis of and experimentation with an SM. When an SM is analysed (the pattern analysing SM (figure 3*e*)), e.g. via a sensitivity or uncertainty analysis, an SM is used as well as potentially some other inputs (X). For example, X may refer to another SE specification when re-using an earlier performed analysis. The result is some SD, e.g. the computed uncertainty and sensitivity characteristics, and an SE specification, e.g. a script or description that allows the analysis to be repeated. The pattern calibrate SM (figure 3*f*) for model calibration is similar, but requires an additional input: some D or an R that serves as the calibration target. A calibrated SM (M’) is produced as an additional output. The pattern validating SM (figure 3*g*) is defined in a similar way to the calibration pattern. The only difference in the pattern is that validation does not produce an SM. For validation, the model behaviour is compared with the D or R and the results are stored as SD.

Compared with Wilsdorf *et al*. [15], we added one additional pattern: *identifying RQ* (figure 3*h*). Newly identified RQs are often a major driver of long-term simulation studies—as well as important results in and of themselves. For example, the modelling work may identify gaps in the data, that lead to RQs for data collection efforts, or the collected data may show interesting properties that pose new questions. In general, any entity (or combination of entities) might lead to new questions. Hence, the pattern for *identifying RQ* allows any input (X) to produce an RQ.

### 2.3. Primary data collection

PD collection includes the design of a collection procedure, the execution of the collection procedure to gather data and the analysis of the data to gain insights.

**Figure 4.**
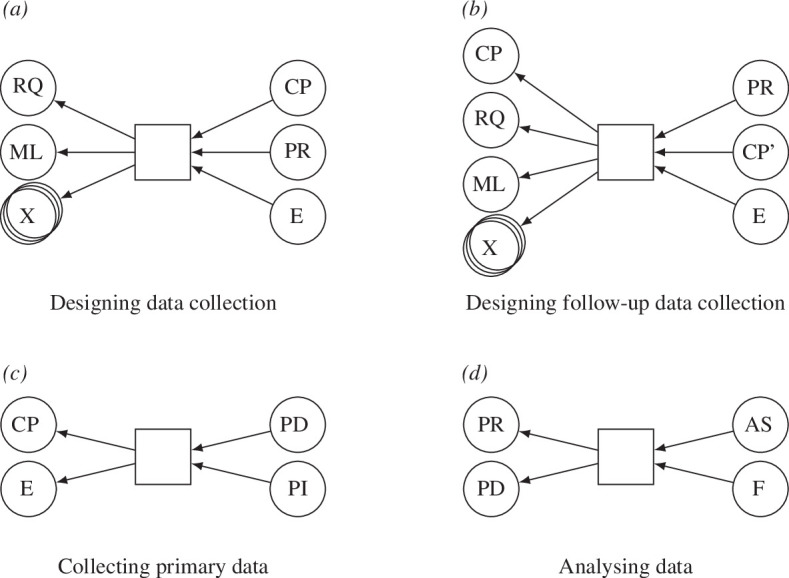
Provenance patterns for PD collection referring to (*a*) designing the data collection, (*b*) designing the follow-up data collection, (*c*) collecting the primary data, and (*d*) analyzing the data. The entity types involved are: RQ, research question; ML, methodology literature; CP, data collection procedure; PR, pre-registration; E, ethical approval; PI, participant information; PD, primary data; F, findings; AS, analysis specification; X, wildcard (here entities of arbitrary type can be added). CP' denotes the follow-up CP as a separate entity. There are two types of relationships: activity used entity, and entity was generated by activity.

We begin by defining the relevant types of entities, and then continue with defining patterns for the activities:

- **methodology literature (ML):** when designing a data CP, researchers often rely on re-using or adapting methodologies from existing research. By including information about key papers that have informed the data CP, other researchers are better able to understand, reproduce and assess the data CP, as well as the PD and findings that are generated;
- **data CP:** the data CP determines what data will be collected and how. Depending on the form of data collection, e.g. a survey, interviews or psychological experiments, this entity may take different forms. For example, it might be a questionnaire, interview questions and instructions for the interviewer, or even a piece of interactive software that is presented to participants. In any case, when presented to the participants, the data CP allows data collection to be undertaken;
- **participant information (PI):** to allow for the assessment and reproduction of PD collection, it is crucial to provide information about the participants included in the study. This includes information such as which populations they were recruited from, how they were recruited and any specific requirements or exclusions that were used (e.g. language requirements, demographic characteristics, attention check questions etc.). Providing this information also allows other researchers to assess the PD collection (e.g. whether the participants were appropriate to address the RQ and support the findings) and to decide whether the data and/or findings are appropriate for other researchers to rely on or re-use (e.g. if they can be transferred to a new population of interest);
- **pre-registration (PR):** pre-registration is a document outlining several key aspects of a study methodology and analysis plan. Some of the key details included within pre-registration are: the specific RQs and/or hypotheses about mechanisms to be explored, the methodology that will be used (e.g. dependent variables and independent variables/experimental conditions), the participant sample size to be collected along with exclusion or inclusion criteria, and the planned analyses that will be used to answer the RQs/test the hypotheses (for an example, see the Open Science Framework registry^1^);
- **ethical approval (E):** PD collection from human participants, be it through interviews or psychological experiments, requires adherence to ethical standards that are set by the funders and institutions carrying out the data collection. Here, the E refers to the final version of the research ethics application, approved by the relevant body, which documents the interview/experiment schedules (data CP), and PI and consent forms, which sets out the conditions and standards of data collection, storage, use and re-use;
- **PD:** the data are the principal result of PD collection. Depending on the kind and scope of data collection, it may take the form of a table or a set of tables, interview transcripts or summaries (excerpts, codes) or a database. In any case, this entity is a representation of the raw output of the data collection, potentially anonymized or pseudonymized if necessary, that may then be analysed in further steps;
- **findings (F):** the F refer to the key conclusions or results generated by analysing the data. These can take a variety of formats, such as a written results section, graphs, tables or descriptive statistics (e.g. means, medians, correlations etc.). These F can subsequently be used as inputs for modelling activities in a variety of ways, including to set or inform model parameters, to help specify the direction of relationships between model variables or to test the broader implications of F (e.g. how a masking intervention influences disease spread through a societal network); and
- **analysis specification (AS):** to reproduce the F, it must be possible to repeat the analysis of the data precisely, either with the same data or with comparable data, e.g. from a follow-up study. Hence, a specification of the conducted analysis is required. Often, the analysis will be conducted with some statistics programming language or library, e.g. in R or in Python. In this case, analysis scripts are a natural result of the analysis process and will allow for easy analysis repetition. If such scripts do not exist, e.g. if an analysis software with a graphical user interface is used, or if the scripts are not sufficient on their own, the specification of the analysis may also be textual. This is similar to the analysis of SD, which is currently made explicit via the SEs. These contain scripts for running the simulations and analysing their output (see figure 3). However, the corresponding analysing data pattern differs from the analysing SM pattern as, for instance, the pre-registration document needs to be considered during PD analysis, and no SM is involved (see figure 4).

We identified the following patterns for the PD collection activities (see figure 4):

- **designing data collection:** before any data can be collected, the data collection process must be designed. This process requires an RQ and methodology literature as inputs, but can also have other inputs (X). One example of a potential additional input (X) is the literature from the substantive area of relevance. We identify three core products of the design phase: the data CP, the PR and the ethics document;
- **designing follow-up data collection:** some data collection efforts are designed to follow up on a previous one, e.g. to replicate the result, to refine the procedure or to answer new questions raised by the F. In this case, the data CP of the original experiment is an additional input when designing the follow-up experiment. Including the original data CP as an input connects the follow-up data collection to the original data collection and shows how the data CP has been refined across multiple rounds;
- **collecting PD:** once the data collection is designed, the data can then be collected. Participants are recruited, and the data collection is executed with them, e.g. they are given the survey or are interviewed. This process is based on the previously designed data CP and must conform to the E. Hence, both are inputs to this activity. The product is the collected data (PD), as well as information about the recruited participants (PI); and
- **analysing data:** when the data are collected, it must be analysed. Apart from the data (PD), the PR, containing the planned analyses, is an input for this activity. The activity produces two outputs: the F, and the AS. While the PR contains plans for the analysis, the actual analysis may still differ from it, especially in the case of exploratory studies or if unexpected issues emerge (e.g. parametric analyses are not appropriate so non-parametric analyses are used instead). Researchers may also wish to explore additional RQs or test the robustness of their results by using additional unplanned analyses. Changes to analysis are perfectly understandable and often recommended, but there must be a clear delineation between pre-planned confirmatory analyses and exploratory analyses. As it only becomes apparent what is actually analysed—and how it is done precisely—during the activity, the AS is produced as part of this activity.

Considerable variation exists in how these practices have been adopted by different research fields, subareas, laboratory groups and even across different studies by the same researchers. For example, although there are many advantages to PR [23], it is not a mandatory practice and therefore may not always be present within a PD collection process. Similarly, although the PD and AS entities are always generated from a PD collection process, these are not always made publicly available or even shared with other researchers upon request (see [24] about the low response rates of authors to data requests). Nonetheless, the inclusion of as many of these entities as possible within a provenance model greatly increases the ability of researchers, including those who conducted the PD collection, to assess the robustness, reliability and relevance of the collected data as well as any findings that were generated. This also has important flow-on effects for subsequent modelling activities that incorporate and rely on the PD. For example, further assessment, new data collection and/or new information coming to light (e.g. failed replications [19]), may lead to the reliability and robustness of a PD collection process being called into question. If this PD collection has been incorporated within a provenance model(s), then researchers can quickly and easily discover which further processes relied on or built upon the questionable PD collection. This makes it much easier to discover and re-examine or re-assess whether subsequent pieces of work need to also be updated or adjusted in light of the questions raised about a PD collection process or output.

### 2.4. Secondary data collection

Unlike PD, secondary data are typically more generic—it does not need to be collected for a specific study. Still, such data can of course be useful for modelling. However, to judge the quality of secondary data, it must be assessed based on relevant criteria for the simulation study. Based on the results of the assessment, the data might then be used as are, or might require cleanup or transformations to address the shortcomings.

For the process of secondary data collection, we have identified the following three entity types:

- **assessment framework (AF):** the AF defines the criteria of the data assessment, dependent on the specific simulation study. For example, the criteria for our case study were specified in Nurse & Bijak [16]. Therein, a set of criteria is defined (such as fitness for purpose, trustworthiness, level of disaggregation, timeliness, completeness, accuracy and so on). There are five levels of evaluation for each criterion, ranging from ’green’ where a desirable criterion is met in full, through ’amber’ when it is met in part, to ’red’ where this criterion is not met (e.g. [25]). In-between ratings (green–amber and amber–red) can also be included. Some criteria are general in nature, determining the extent a given source may be useful, whereas others are linked to the bias and variance inherent in the data source, which needs to be considered for the modelling process;
- **metadata (MD):** MD are properties of the dataset in question, including source, a short description, a URL, time details, source type, topic, data types, as well as the values of specific evaluation ratings from the AF given to the data source; and
- **cleaned data (CD):** a product of transforming the initial data (D) taking into account their properties (MD), aimed at creating new variables with desired properties, such as being devoid of explicit bias or having reduced variance. A migration-related example can be: if migrant registration data (D) are known to be under-reported (one of the properties of MD, completeness, is rated ’amber’, indicating a presence of bias), then CD can include daily rates of change in registrations rather than volume of registrations because the former would be less sensitive to the presence of systematic bias.

**Figure 5.**
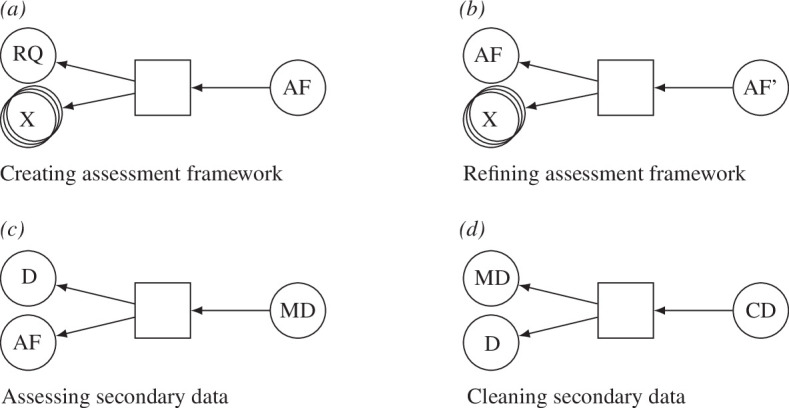
Provenance patterns for secondary data collection referring to (*a*) creating an assessment framework, (*b*) refining the assessment framework, (*c*) assessing the secondary data, and (*d*) cleaning the secondary data. The entity types involved are: RQ, research question; AF, assessment framework; D, data; MD, metadata; CD, cleaned data; X, wildcard (here entities of arbitrary type can be added). AF' denotes the refined AF as a separate entity. There are two types of relationships: activity used entity, and entity was generated by activity.

The following four patterns for secondary data have been also identified (figure 5):

- **creating AF:** as the AF is specific to the simulation study, its creation is the necessary first part of the assessment process. The connection to the rest of the study is realized by using the RQ as input. Other inputs (X) may include, but are not restricted to, earlier AFs or knowledge about limitations of the data relevant in the field. The product is the AF;
- **refining AF:** at some point during the study, the existing AF may need refinement, e.g. when the RQ has shifted enough that the previously defined criteria no longer fit. This activity uses the previous AF, as well as potentially other sources (see ‘creating AF’). It produces a refined AF. To distinguish the two versions of the AF, the refined version is denoted as AF’ in figure 5*b*;
- **assessing secondary data:** the assessment of some data is the application of the assessment framework to that data to determine the properties of the data. Hence, the AF and the data (D) are used by the activity, while the MD are produced; and
- **cleaning secondary data:** the transformation of the data (D) in the light of the data properties (MD) identified during the process of applying the AF, in order to produce CD. The process may involve steps such as removing the identified biases, smoothing data to reduce variance, applying a variable transformation to reduce other issues identified in the assessment process (such as log-transformation for strictly positive variables which exhibit exponential patterns of change) and so on.

There are existing frameworks for comprehensively assessing the different aspects of the quality of data according to different criteria (e.g. [25]). The inclusion of data assessment in a provenance model not only ensures that quality checks and corrections can be formally embedded as a necessary element of the modelling process, but also enables identifying which parts of the model may be affected by potential problems with a particular data source (e.g. [26]). This makes the ensuing modelling and analysis explicitly conditional on the information used and data cleaning activities undertaken. It also means that, where needed, uncertainty from the data can be propagated to the model results along the paths of the provenance graph, helping with analysis transparency and with honest reporting of the results and their limitations. Alternatively, the provenance sub-graphs related to data analysis and cleaning (figure 5) may describe a piece of analysis in its own right, should data-related question be of specific interest to the analysts or the users of a particular data source.

### 2.5. Agents

The human or software actors responsible for conducting the various parts of a simulation study may also be added to the provenance graphs. Note that in PROV these are named ‘agents’—not to be confused with the agents implemented in the agent-based SM.

In the visual representation of PROV, agents are typically depicted as hexagon, diamond shape or similar. They can be used to express who is the owner of an entity (*attribution*), who is responsible for and what role they played in an activity (*association*) and who assigned responsibility to an agents (*delegation*). Consequently, including agents may be beneficial for simulation studies to increase the traceability and reproducibility of a study.

Incorporating agents explicitly as part of provenance meta-information is also crucial for improving the trustworthiness and interpretability of a study. Literature on modelling in the social sciences underlines that who was involved in the various modelling and analysis activities matters, given the diverse decisions researchers make. For instance, in a study about model variability, modellers exhibited differences in the formalization of cognitive theories, employing different levels of abstraction, including different factors in their models, and even using different data as input [27]. Furthermore, the creation of diverse agent-based models of empirical systems is recognized as an own research methodology [28]. Specifically, the exploration and comparison of diverse perspectives on a system through the use of alternative models can produce valuable insights for management decisions. Referred to as model-to-model analysis, this approach allows us to determine which model and perspective best align with empirical data [29].

Besides human agents, according to PROV, the term may also refer to software agents. In social simulation studies, these may be, for instance, open databases that regularly provide new datasets, or an automatic experiment generator that can (more or less) autonomously generate and execute new SEs [15].

Reporting documents, such as TRACE and ODD, may also include information about the used software, and who was responsible for collecting data, or developing a sub-model [1,6]. However, provenance has the unique ability to provide a fine-granular mapping between entities, activities and agents over time.

## 3. Proof-of-concept

To demonstrate the approach, we realized a provenance model of route formation in asylum migration from Syria to Europe. In terms of software, we extended WebProv [8]. It allows for the creation and editing of a provenance graph of simulation studies with a web-based interface. For brevity, the nodes in the following provenance graph are labelled with two or three letters followed by a number, e.g. RQ1 for the first entity of type RQ. WebProv, however, also allows for custom labels to be set, offering a more intuitive option for the broader audience of provenance graphs.

In the case study, activities such as model building, data collection, etc. were conducted by a group of people, and each cooperation partner had different responsibilities. However, in the following, we withhold the agents for reasons of readability as the provenance graph is already rather complex.

The provenance graph is stored in a graph database (Neo4j), which not only allows for simple and efficient storage but also includes a powerful language for retrieving information from the database. Documents and artefacts referenced in the meta-information are stored online in appropriate repositories, e.g. on GitHub, the Open Science Framework (OSF) or Zenodo. Our extended version of WebProv and the provenance graph presented here are available in Zenodo archives at Reinhardt *et al.* [30] and Reinhardt [31].

### 3.1. Developing an agent-based model of migration route formation

Migration is a highly complex and uncertain population process, driven by the decision-making of individuals and various institutions. Migration routes are highly volatile, with the flows responding to changes in various migration drivers, broader environments and individual circumstances, which can sometimes change rapidly [32]. In this case study, agent-based simulation is applied to improve the theoretical understanding of human migration, with a specific focus on the question of how migration routes are established and sustained.

The core of the study [21] is the development of an agent-based model of migration route formation [33] in a domain-specific modelling language with fast continuous-time execution [34]. Therein, modelled migrant agents attempt to traverse an abstract landscape based on limited and uncertain information about locations on the way, potential paths and the involved risks. As model development is an iterative process [35], multiple model versions were designed in succession, informed by knowledge from the scientific and non-scientific literature on the migration process, knowledge about decision-making and lessons learned from previous iterations. For the latter, extensive SEs with the model were necessary. To that end, at each step Gaussian process emulators were fitted to the model outputs of each model version to assess sensitivity to the input parameters and the uncertainty of the results. While earlier model versions were very abstract and theoretical, later versions were designed and calibrated to capture the reality of migration routes in the central Mediterranean. Thereby, a considerable amount of data were integrated into the model.

Data in general, especially migration data [26], tend to be difficult to compare and may sometimes be incomplete or of dubious quality. Hence, an important part of the project was assessing available data on asylum migration. To this end, an AF was designed and applied to various potentially useful sources of migration data [16], so that the data were supplemented with the necessary meta-information about quality to enable the use of the data in the simulation study. This migration data from secondary sources were also complemented with information on the migrants’ decision processes, elicited as PD through psychological experiments and interviews which were designed to answer specific questions that arose during the modelling work. For example, the sensitivity analysis of earlier models highlighted information sharing and trust in information as key influences in forming migration routes. Subsequently, in a bespoke psychological experiment, data on migrants’ subjective judgements based on different kinds of information and sources were collected. The results were then used to inform the parameterization of the successive model versions [36].

The migration case study highlights that simulation studies of complex social systems are themselves complex and intertwined processes that are conducted by an interdisciplinary group of researchers and include the modelling work itself, the execution of SEs, the collection and assessment of secondary data sources, and the collection of new data to inform the model. Broader philosophical underpinnings of such a model-based approach, within which the iterative model development is situated, are discussed in more detail in Bijak [21, ch. 2].

These diverse modelling activities, data and all of the used information sources are dependent upon one another and contribute to the products of simulation studies. Each of these products can only be properly interpreted if their generation context is fully taken into account. Therefore, accessible and thorough documentation of simulation studies becomes of utmost importance.

### 3.2. Provenance overview

**Figure 6.**
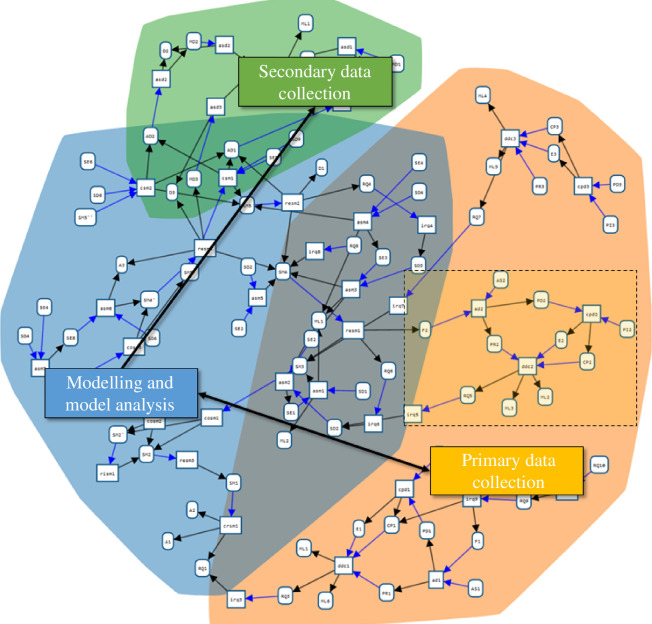
Overview of the case study provenance graph in WebProv. Each node is associated with one part of the project, as distinguished in this paper: the modelling and model analysis (blue), the PD collection (orange) and the secondary data collection (green). The subgraph in the dashed box is detailed further in figure 7.

On the whole, the data for the presented model of migration came from a range of primary and secondary sources [21]. Figure 6 shows an overview of the provenance graph as a whole. Examples of PD include psychological experiments on human decision-making (three shown), later supplemented by ethnographic interviews [37]. Secondary data include administrative or survey-based statistics as well as qualitative information on the known numbers of arrivals, interceptions and fatalities, as well as potentially relevant aspects of migration journeys themselves, such as frequency and modes of communication with others and trust in information sources. A complete listing of secondary sources considered for this modelling exercise, together with their basic meta-information and quality assessment, are available in Nurse & Bijak [38].

**Figure 7.**
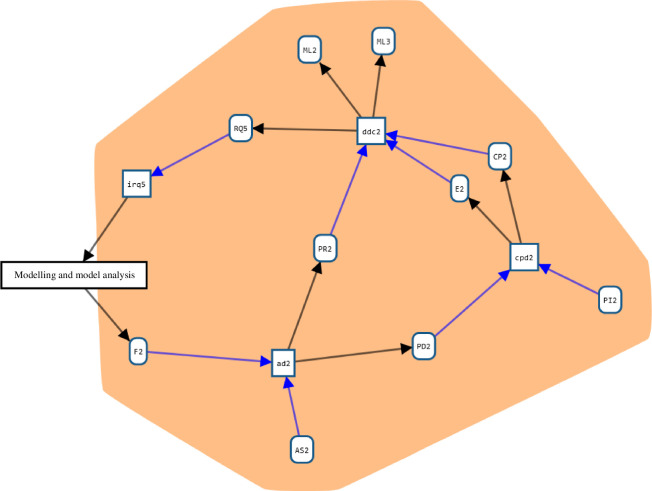
Detail of a part of the PD collection: the experiment on subjective probability judgements. The box on the left labelled ‘modelling and model analysis’ refers to that part of the project (the blue area in figure 6). Arrows pointing to or from it represent provenance relations (‘used’ or ‘was generated by’) with nodes in the ‘modelling and model analysis’ part. In WebProv, this box may be ‘opened’ to display the actual relevant nodes.

**Figure 8.**
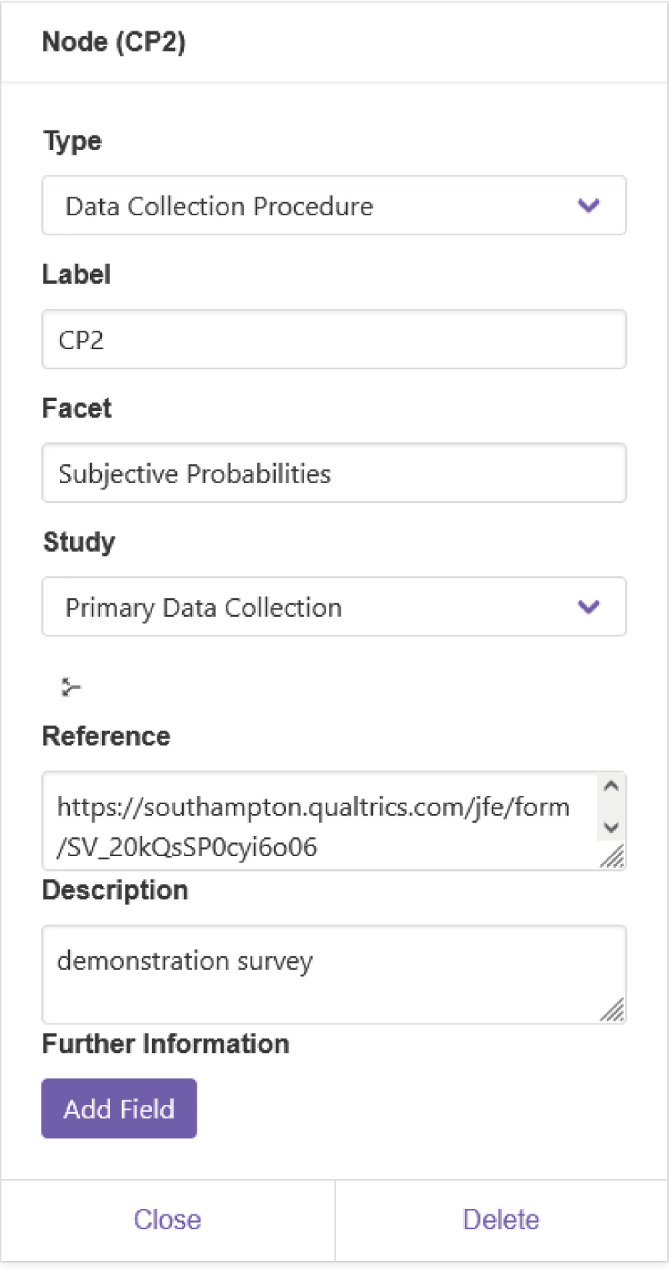
Information about the entity CP2, the data CP of the psychological experiment described in the text, as displayed in WebProv. The field ‘Facet’ allows a keyword to be entered, to aid in searching for related entities, e.g. all entities related to this experiment (see figure 7) have the facet ‘Subjective Probabilities’. The ‘Study’ field refers to the different coloured areas, which are called studies in WebProv. ‘Reference’ contains a reference to the collection procedure itself, in this case, in the form of a demonstration survey identical to the one given to the participants. The ‘Description’ field allows additional information to be summarized.

Figure 7 shows a part of the provenance graph in greater detail, in particular focusing on an instance of PD collection: a psychological experiment to elicit subjective probability judgements migrants make based on information they gain from different sources. The full experiment and its results are documented in Prike *et al.* [39]. While the figure only shows the graph, the interactive user interface displays detailed information about each entity and activity when it is selected (see figure 8) (often giving a concise description, some key properties and referencing the document or piece of software represented by an entity). In the activity irq3, an RQ is identified (see the pattern *identifying RQ,* figure 3*h*), based on some entities in the ’modelling and model analysis’ area that are not displayed here. Starting from this question, a psychological experiment was designed (*design data collection*, ddc2), using ML2 (referring to Briñol & Petty [40]) and ML3 (referring to Wintle *et al*. [41]) as ML inputs—one satisfying the ML input of the pattern, and the other serving as an optional additional input (X). The results of this activity are a data CP (CP2; referring to the survey^2^), the PR (PR2; linking to the PR stored on OSF^3^) and the E (E2; referring to the University of Southampton Ethics Committee, ERGO number 56865). Similarly, cpd2 and ad2 match the patterns *collecting PD* and *analysing data*.

As demonstrated, the provenance graph can serve as a high-level overview of the various activities of a simulation study, connecting the various inputs and outputs. For large-scale studies with many interconnected parts, the graph will become increasingly large and complex, reflecting the complexity of the documented study. However, the semi-formal structured approach allows for computational processing of the provenance graph, as we demonstrate in §3.3.

### 3.3. Retrieving detailed information: querying

The provenance graph not only gives an overview of the conducted simulation study, but it is also rich in detailed information, linking various artefacts produced in the study. Querying allows for the retrieval of detailed information on demand. Using a dedicated graph database for storing the provenance graph, we can exploit the included querying language, in this case, Neo4j’s Cypher, to formulate graph queries effectively and have them executed efficiently. In practice, retrieving some detail requires two steps: first, a Cypher query must be formulated and executed to retrieve the provenance nodes of interest and the relationships between them. Second, the meta-information of the nodes of interest may be inspected interactively, either for the information itself or to follow references to the relevant documents. For this, our tool WebProv offers simpler queries of individual entities (i.e. by their name and other attributes), and means for zooming in and out of the area of interest. In the following, we show some typical questions that may be asked of a simulation study about the RQs, the model building and the relation to data, and demonstrate how they can be answered with queries on the provenance graph.

Often not only single entities, but their context within the study is of interest—after all, putting the entities into the context of their generation and use is the point of provenance models. For example, we might want to ask for RQs that were newly asked within the study—and what they are based upon. This context can be specified in the query as a graph pattern:


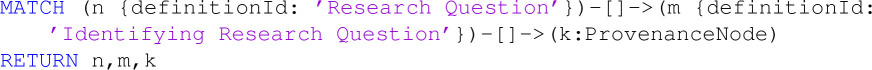


**Figure 9.**
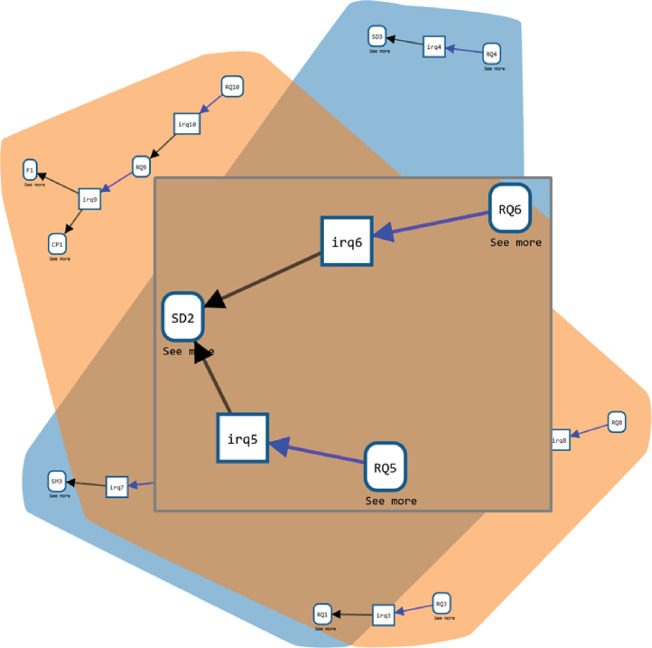
Result of a query for RQs identified in the project. The grey box shows a part of the result: RQ5 (‘how do migrants make likelihood judgements?‘) and RQ6 (‘how do risk perception and risk avoidance affect the formation of migration routes?‘) both follow from SD2, the result of an analysis of the model that shows that the parameters related to risk are most sensitive, requiring more research on this subject, and hence the two new RQs. The background shows the complete result of the query.

Here, we query for all RQ entities n, the identifying RQ activities that generated them m and any entities k that were used in these activities. The result is displayed in figure 9. Please note that the initial RQs of the study are not displayed, as we specifically asked for RQs generated within the study. In this particular example, the query allows for identifying those experimental results (RQ5 ‘how do migrants make likelihood judgements?‘) that correspond to the mechanisms underpinning the model assumptions on decision making (RQ6 ’how do risk perception and risk avoidance affect the formation of migration routes?‘). This enables incorporating experimental findings into the model, along with identifying knowledge gaps that need filling through further data collection.

The same approach also allows for the querying of complex graph patterns, i.e. asking questions about relationships between entities and activities of the simulation study. One might want to know how a certain finding from a psychological experiment, e.g. the findings of a psychological experiment on the subjective judgement of migrants concerning different kinds of information and sources (the entity labelled F2), influenced the SMs. In terms of the provenance graph, this means asking for SMs from which a path (possibly via multiple intermediary steps) leads to F2, as well as for the nodes on this path:


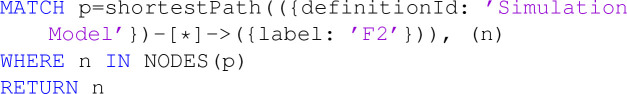


**Figure 10.**
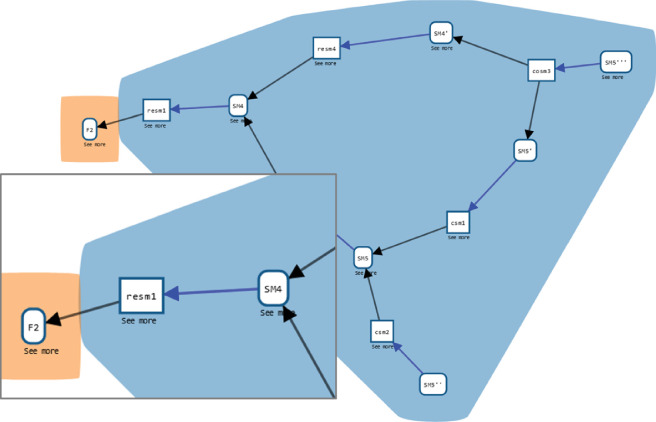
Result of the query for models that use the findings of an experiment on the subjective judgement of migrants on different kinds of information and sources (*F2*). The findings were used in creating the model version SM4 (see the enlarged box). Through SM4, they also influenced later model versions. If new conflicting findings emerged, the preceding model SM3 could be used to develop an alternative SM to SM4. Thus, model families might emerge that share a core but allow for exploring different scenarios based on different findings.

Here, we use the shortest path, to only see the most direct path from any SM, hiding more indirect relationships. The result of the query, a sub-graph of the provenance graph, can be seen in figure 10. This shows the empirical findings that informed the building of SM4, as well as later model versions via SM4. In this way, the new version, incorporating the experimental results, transformed the model from being a mostly theoretical exercise to becoming grounded in the empirical evidence about decision making, in this case, on the perceived risk of making a migration journey and how it varied depending on the information received from various sources [21].

As a final example, one might be interested in how the modelling work was grounded on the other work conducted in the study, e.g. on the collected data. In the query, we are looking for any links from nodes in the ’modelling and model analysis’ area to other areas of the study. For the sake of clarity, we only want to display the first entity or activity outside of the ’modelling and model analysis’ area:


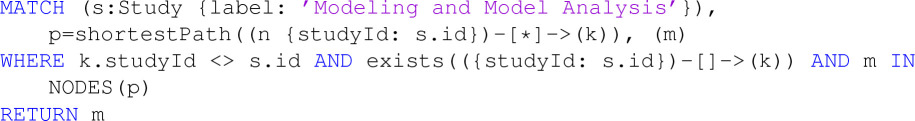


**Figure 11.**
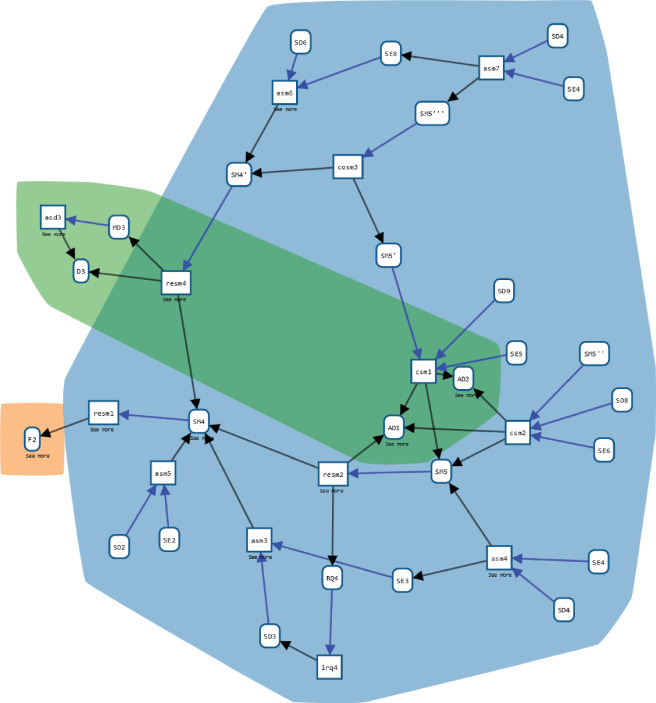
Result query for sources of SMs outside the ‘modelling and model analysis’ area. The node on the left is F2 (as in figure 10), the nodes in the green area are from the ‘secondary data collection’ area. The amount of external, different sources might indicate how many research results are brought together by the model and, thus, its integrative quality.

These areas are called ’study’ in WebProv (which is a different use of the term study than elsewhere in this paper). We identify s as the WebProv study ’modelling and model analysis’. Then, we search for paths from a node n within the WebProv study s to other nodes k that have a different WebProv study id, i.e. are in a different area. Further, these nodes shall have a predecessor with the same WebProv study id as n, i.e. which is in the same area as n. Figure 11 shows that the modelling work was grounded on the F2 findings from the PD collection as well as several entities from the secondary data collection, leading to a more developed and fine-tuned version of the model. The provenance query may also be refined, e.g. to ask specifically about the secondary data.

## 4. Comparison with existing approaches

The wish to reproduce, interpret and re-use the results of simulation studies has led to various forms of computational support for recording crucial information about simulation studies. These include adopting archives [42], wikis [43], electronic notebooks [44], as well as reporting guidelines in different application fields [3,45].

Some reporting guidelines focus on specific activities or products of a simulation study. For example, there are guidelines focused on the SM, e.g. minimum model reporting requirements, preferred model reporting requirements for systems dynamics models [3] or ODD for agent-based models [6,20,46]). There are also guidelines for SEs, e.g. minimum information about a simulation experiment (MIASE) [47], minimum simulation reporting requirements (MSRR) and preferred simulation reporting requirements [3]. These reporting guidelines are not intended for describing collections of different artefacts nor the process of entire simulation studies. In the case of ODD, Grimm *et al*. explicitly state: ’An ODD corresponds to the ’Materials’ part of the ‘Materials and Methods’ section of a scientific publication because it describes the virtual laboratory in which we conduct SEs. The ’Methods’ equivalent then must describe how we used the materials—the model—in SEs. Previous publications on ODD recommended that an ODD be followed by a section entitled ‘Simulation Experiments’ but provided no further guidance’ [6, § 5.6]. They also point out that: ‘There certainly is also the need to describe a model’s underlying story, or narrative, but ODD is not the place for this [...]’ [6, § 3.5].

The most closely related approaches to ours include TRACE [1] and STRESS [2]. These aim at documenting the entire simulation study and thereby covering all of the essential steps, sources and products of a modelling and simulation life cycle [48,49]. Similarly, the XML-based documentation format by Triebig and Klügl contains several elements that refer to the entities RQ, requirement, SM and SE of our approach [50]. However, Grimm *et al*. state the limitation of these approaches and particularly TRACE: ‘TRACE, though, is a format for supplements, not for the main text. What might be needed is a format corresponding to TRACE but which is suitable for main texts’ [6, § 5.8]. Provenance graphs based on PROV, on the other hand, have been shown to be a viable option to achieve this goal. In the publications of Haack *et al*. [9,51], we included provenance graphs that document the entire simulation studies within the ‘Methods’ section of the main text.

The benefits of applying PROV to make provenance information queryable have been discussed by Pignotti *et al*. who suggest that documenting provenance in a simulation study refers to one of three things [52]: (i) the process of model development, (ii) the execution parameters of the model, and (iii) history of a simulation run. Pignotti *et al*. focus on (iii), whereas our approach is clearly linked to (i). Bennett *et al*. do not apply the PROV standard but are also concerned with type (iii) provenance. They specifically underline the importance of capturing provenance information about state transitions and cause–effect relationships between input and output of a simulation [53]. Mitchell *et al*. propose PROV as one possible means for representing the provenance of data pipelines including used software and initial model configurations [54]. They therefore partly refer to provenance type (ii).

As central novelty, our approach presents PROV patterns to structure and model the process of conducting a complex simulation study. Unlike previous approaches, our paper outlines an approach that allows for the inclusion of various versions of SMs, the calibration and validation of those models, and interweaves this process with the acquisition of original and secondary data, as well as the cleaning and inclusion of those data within the SMs. This approach allowed us to represent a 5-year research endeavour in a manner that enables easy access and querying of the origins and dependencies that the different results stem from and rely on, providing credibility and traceability for the entire simulation study from beginning to end.

**Figure 12.**
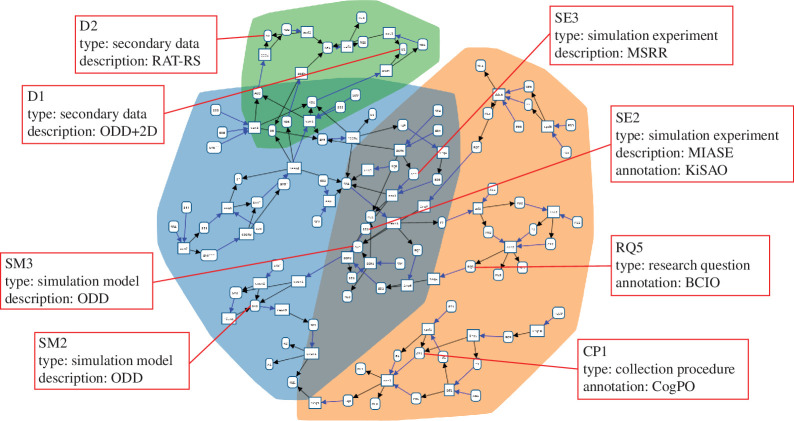
Exemplary usage of reporting guidelines and ontologies for describing and annotating the entities of the case study. For instance, if a SM entity in the provenance graph is given by an agent-based model, an ODD [6] document may be linked in the meta-information of this entity that describes this particular model (e.g. SM2 and SM3). Another example is the reporting standard MIASE [47], which may be used to describe the SEs (e.g. SE2). In addition, the ontology KiSAO [58] may be used to unambiguously annotate which simulation algorithm was used in the experiment.

Provenance patterns can and should be combined with the aforementioned documentation guidelines for describing the meta-information or attributes of the entities. Semantic annotation of provenance using ontologies can add domain-specific meta-information, which is crucial when reasoning, e.g. about the cognitive paradigms used in psychological experiments (cognitive paradigm ontology (CogPO) [55]), the expected effects of interventions on human behaviour (behaviour change intervention ontology (BCIO) [56]) or the modelling methodologies applied (discrete-event modelling ontology [57]). Further ontologies for the social sciences can be inspired by the various ontologies of bioinformatics, e.g. the kinetic simulation algorithm ontology (KiSAO) for simulation procedures [58]. Figure 12 highlights the relation of our approach to existing standards and ontologies for documenting RQs, data, data CPs, SMs and SEs using the migration case study as an example. As described above, we do not aim to replace documentations—all documentations have a unique purpose and may (and ideally should) be used in combination. Depending on what information is required, e.g. about the workings of the agent-based SM (ODD), about which model used findings from a specific psychological experiment (PROV), or about which approach was used in a psychological experiment (CogPO), one or the other documentation can be consulted. In addition, meta-information should be combined with accessible and executable versions of SMs and SEs in accordance with the findable, accessible, interoperable, reusable (FAIR) principles for open data [59]. Thus, each entity of type SM or SE should refer to an openly accessible code repository. The accessibility and transparency of code can be enhanced by exploiting developments and standards in specifying executable SEs, including domain-specific languages [60–62] or model-based experiment designs [63].

Whereas documentations based on the various reporting guidelines assume one purpose and one single SM, we assume that different versions of SMs and even RQs can belong to the documentation of a simulation study. The provenance graph can be seen as a ‘meta-model’ (a term used here in a different sense than in statistics and uncertainty quantification, see Bijak [21] for discussion), describing the generation process of a SM in terms of activities such as model creation, refinement and composition, as well as the generation of RQs and their inter-relationships with sources and (intermediate) products of the simulation study. Our approach’s unique perspective on the story of simulation study also becomes evident when we look at activities such as model analysis, calibration and validation.

Furthermore, we make a unique contribution toward specific aspects of social simulation studies, including the acquisition and usage of both primary and secondary data in an interdisciplinary modelling endeavour. In all reporting guidelines of simulation studies, information about the used data is required, e.g. in its checklist, STRESS asks for details and purpose of data sources, input parameters for base runs of the model and scenario experiments, assumptions and data pre-processing. The latter refers to any manipulation of the data that occurred. In the TRACE documentation, the data evaluation section should provide insights into the quality and sources of numerical and qualitative data that have been used to parameterize the model. While the use of data is generally included in these documentation standards and sometimes even put into focus, e.g. by the rigour and transparency—reporting standard (RAT-RS) [64] and ODD + decision + data (ODD+2D) [65], the procurement of data, including assessment of data with explicit criteria and data transformation, is usually not. Often, the approaches for documenting and recording information can rely on reporting guidelines for data acquisition and generation in the respective application field. In the social sciences, this may include various types of quantitative and qualitative data, such as data from psychological experiments, interviews, surveys or official sources. The replication crisis in psychology, and the subsequent focus on uncovering questionable research practices in psychology and empirical research more broadly, led to the development of several suggestions and guidelines for how to document and improve rigour, openness and transparency in empirical research [19,66]. For PD collection, these practices include: making collected data and analysis code publicly available, publicly posting the study materials and procedure, being transparent about the ethical aspects and PR study protocols and analysis plans ahead of time [67–69]. Although some of these practices are not directly applicable to secondary data collection and analysis, practices such as sharing analysis code and clearly specifying analysis plans ahead of time are also strongly recommended for improving the transparency and rigour of research relying on secondary data [18,70]. However, there is still a long way to go before these practices become standard.

The use of provenance models such as PROV to document the process of data collection, transformation and analysis has been explored in the context of data science [71] and scientific workflows [72]. There, the provenance of data is dissected into more fine-grained steps, i.e. the individual commands executed via a script. By contrast, our approach represents data provenance on a unique level of abstraction, focusing on the central macro-level activity patterns involved in the process of primary and secondary data acquisition.

## 5. Conclusion

In this paper, we introduced provenance graphs based on the PROV standard as a means for explicitly and concisely telling the whole tale of a social simulation study. The definition of provenance patterns has enabled the representation of key entities, activities and the specific relationships between them. We have also presented a tool based on modern web technology for creating and exploring these comprehensive provenance graphs.

The documentation approach presented in this paper differs from existing documentation guidelines for simulation studies such as TRACE or STRESS in three key aspects: scope, subject and degree of formalization and computational accessibility.

Unlike the aforementioned guidelines, we treat primary and secondary data collection as an integral part of the simulation study. To judge the foundations of a SM, it is not enough to just know that data were used. The quality and suitability of data must also be assessable. Unlike TRACE [1] or STRESS [2], which are primarily concerned with what data were used and where it is used in the modelling process, the provenance approach presented in this paper makes the collection of PD and the assessment, preparation and cleaning of secondary data explicit. The detailed documentation of both collection procedures (for PD) and assessment criteria also aims to greatly improve the visibility of the limitations of the data.

The provenance graph focuses on documenting a simulation study’s processes, not its products. The provenance patterns we suggest do not describe a SM, SE or piece of data. Instead, they describe how they were created, what steps were undertaken and how they relate to the specific RQs they were designed to answer, to data they were based upon and to the results they generated. Our approach may also include activities such as failed calibration and validation attempts, which would otherwise not be reported. It thereby enhances the traceability of individual modelling decisions. Consequently, the provenance graph is not intended to replace other documentation but to complement it. The whole graph models the process of the study. Single entities document individual (intermediate) products, for which existing documentation standards can be employed. For instance, the ODD protocol [6,46] targets the documentation and communication of a single SM, whereas MIASE [47] should be employed for describing individual SEs.

Unlike most documentation standards and protocols used or suggested for social simulation, which are textual, we propose a semi-formal approach that stores information within a graph structure with partly formalized meta-information. This aims to make the documentation more accessible for computational processing.

We demonstrate some of these benefits in §3 by using graph queries to retrieve information about the simulation study. For instance, using a single query, we could identify what primary or secondary data the different modelling steps were based on. Moreover, based on queries, the relationhip between simulation studies can be analysed, as done by Budde *et al*. [8].

The benefits of provenance graphs and patterns are not restricted to the typical use cases for documentation that generally only occur after the simulation study has been completed. Wilsdorf *et al*. [15,73] demonstrate how the provenance graph can automatically generate new SEs for new model versions while modellers are conducting their simulation studies, thereby reducing the time and manual effort required. Moreover, automatic processing and conduction of simulation studies will be essential to help remedy the various methodological challenges of computational social sciences [74]. Some of these challenges involve the accurate and appropriate use of statistics, data science and other modelling approaches across a range of applications and scientific disciplines. By developing a common, formal standard for documenting, visualizing, querying and analysing different stages of the modelling process, provenance models provide a promising approach to overcoming many of these challenges. They make simulation studies and the involved processes intelligible to both model producers and users from diverse areas of science and practice.

## 6. Future work

Beyond the proof-of-concept presented in this work, our objective is to collaboratively refine and expand our methodology together with modellers from different research groups. This endeavour will entail conducting further case studies across the diverse areas within social simulation. Additionally, we need to carefully consider the trade-off between the learning curve associated with adopting the provenance tool and the benefits gained from explicitly documenting provenance in a graph-based format.

To incentivize wider adoption of provenance graphs and provenance patterns in social simulation studies, our approach should ideally be paired with additional documentation guidelines and software.

We aim to further integrate established reporting guidelines for specifying the provenance entities and meta-information. Figure 12 illustrates which purposes the various reporting guidelines can serve within the provenance. The information they provide can come either as verbal narratives, semi-structured elements like parameter tables, or formal elements like equations. We plan to incorporate ontologies to disambiguate and refine the provenance meta-information, such as the specific simulation algorithm used [75].

So far, the provenance graph in our case study was documented manually using the web editor. Since this is a tedious task for modellers, solutions for automatically capturing provenance information are desirable. Automatic non-intrusive provenance capturing is, for example the aim of the NetLogo extension Backtracer [76]. Alternatively, several workflow systems have built-in capabilities to assist modellers in collecting all the relevant provenance information [7,72]. However, this requires the modeller to get acquainted with the respective workflow system; and still, not all the necessary details can be recorded automatically. More transparent systems, that do not interfere with the steps and software environment of the users, are the focus of current research [77].

The insights gained from provenance graphs are constrained by the intelligibility of the query language. However, queries on the structure of a graph are inherently complex. Modern graph query languages (such as Cypher) already consider the trade-off between expressivity and complexity [78]. We plan to equip our web tool with templates that may assist users in specifying recurring types of queries. An alternative user-friendly approach would be to automatically generate such queries from questions formulated in natural language. Here, one could take advantage of new developments in the area of large language models [79]. Queries may also assist in aggregating provenance graphs so that different views with defined semantics can be generated to support the needs of different user groups. The meaningful reduction of provenance graphs is an ongoing research topic [7,80].

Provenance graphs of continuing as well as completed studies can support modellers in conducting their simulation study more efficiently and systematically, especially so in interdisciplinary projects [15]. For instance, the approach by Wilsdorf *et al*. re-uses and adapts information given by provenance (i.e. earlier SEs, data, assumptions and qualitative models) to generate new SEs for calibration, validation and analysis. Further tools like this need to be developed that exploit provenance information to support and automate the various parts of simulation studies.

## Data Availability

Relevant code for this research work is stored in GitHub [81] and has been archived within the Zenodo repository [30]. Data have been archived within the Zenodo repository [31].

## Footnotes

^1^
https://osf.io/3qrs8.

^2^
https://southampton.qualtrics.com/jfe/form/SV_20kQsSP0cyi6o06.

^3^
https://osf.io/3qrs8.
